# Secondary Submuscular Gluteal Implant Replacement: The Safe Hybrid Bridge Technique

**DOI:** 10.3390/jcm14134486

**Published:** 2025-06-25

**Authors:** Mattia Colli, Salvatore Giordano, Enrico Dondè, Alessandro Gennai

**Affiliations:** 1 Podgora7, Private Practice, Via Podgora 7, 20122 Milan, Italy; mattiacolli@shbclinic.com; 2 Department of Plastic and General Surgery, Turku University Hospital, 20520 Turku, Finland; 3 Studio Dondè, Private Practice, Via Senato 25, 20121 Milan, Italy; enricodond@gmail.com; 4 Studio Gennai, Private Practice, Via Delle Lame 98, 40122 Bologna, Italy; agennai@mac.com

**Keywords:** plastic surgery, buttocks augmentation, prostheses, implants, esthetics

## Abstract

**Background**: Gluteal augmentation and reshaping have recently gained popularity due to growing patient demand. The rising number of intramuscular gluteal augmentation procedures has led to a corresponding increase in implants, although this has not reduced noticeable esthetic flaws and relatively common postoperative complications. The patient often opts for a secondary gluteal replacement implant. However, studies on this procedure are scarce. **Methods**: We describe our secondary submuscular gluteal implant replacement technique in patients complaining about dislocation or complications with the primary intramuscular gluteal implant. This procedure involved creating a new round implant pocket in a deeper anatomical plane while keeping the gluteus maximus muscle bridge that joins the intramuscular pocket with the submuscular pocket as intact as possible. To achieve this result, we describe the safest way to remove the intramuscular implant: a small incision is performed posterior to the iliac crest, through which the intramuscular gluteal implant is removed via a small intramuscular-subcutaneous tunnel. We reviewed surgical data, focusing on implant size choice and postoperative complications. **Results**: We performed this technique on 108 patients. The most frequent complications included three cases of laceration of the muscular bridge between the old intramuscular plane and the new submuscular pocket, resulting in both pockets merging, and eight cases with temporary nerve pain lasting a few days postoperatively. **Conclusions**: This study validates our approach for addressing and preventing dislocation or other complications from intramuscular gluteal augmentation procedures with implants by applying a safe technique that involves replacing the intramuscular implant with a submuscular one.

## 1. Introduction

Since its inception in 1970 for esthetic purposes, a diverse array of surgical techniques for gluteal augmentation has emerged [1,2,3]. These include autologous fat grafting, hyaluronic acid injections, methacrylates, poly-l-lactic acid, adipose fascial flaps, and implant insertion [4,5,6,7,8,9,10,11,12,13,14]. Notably, buttock implant surgery has undergone significant evolution, particularly with the introduction of the intramuscular technique [15,16,17,18]. Over time, intramuscular implant placement has gained favor over subfascial and subcutaneous placement due to its perceived advantages as a middle ground between deep and superficial placement [19,20]. However, intramuscular gluteal augmentation with implants has been shown to cause muscle atrophy, even if muscle function was not compromised [21,22,23].

In recent years, there has been a notable increase in the number of gluteal implant augmentations, leading to a corresponding rise in complications such as implant palpability, capsular contracture, hernia, implant displacement, asymmetry, and rippling, which often necessitate secondary surgery [24,25,26]. These complications are influenced by factors such as the anatomical plane of the implant pocket, the type of implant used, and the surgeon’s technical experience [27].

Despite this trend, there is a paucity of studies in the medical literature exploring techniques for secondary gluteoplasty or their postoperative outcomes. Gonzalez briefly outlined the principles guiding secondary operations, Jaimovich reported a case of secondary gluteoplasty, and Serra & Aboudib described gluteal implant displacement and its treatment [27,28,29].

This manuscript presents a technical innovation case series with the primary aim of describing our optimized technique for secondary implant replacement, which we named “The Safe Hybrid Bridge Technique”, as it replaces the implant from an intramuscular pocket to a submuscular one (hence hybrid) while maintaining the gluteus maximus muscle bridge intact. This technique allows the management and prevention of dislocations or other complications associated with intramuscular gluteal augmentation using implants. Moreover, we present the outcomes based on retrospective data collected from 108 patients who underwent the procedure in our private clinical settings, to provide insight into the safety and effectiveness of the technique in real-world practice.

## 2. Materials and Methods

### 2.1. Study Design, Setting, and Patient Population

**Figure 1:**
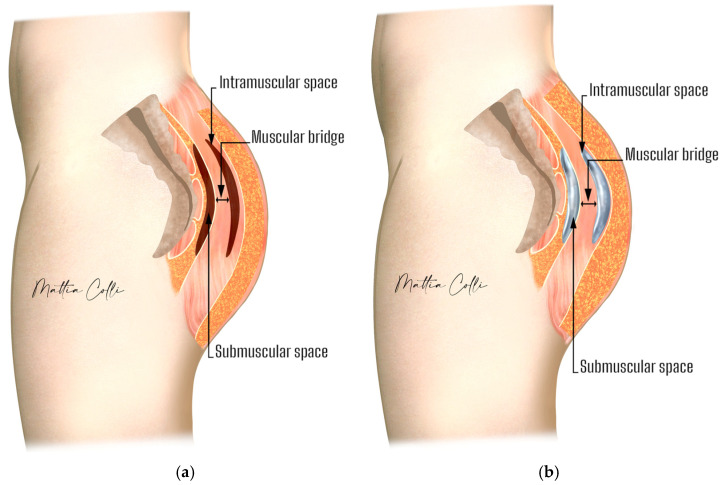
Anatomy of the buttocks. (**a**) Illustration showing the intramuscular and submuscular space. (**b**) Implant placement. The green sign shows the muscular bridge.

We conducted a retrospective observational analysis of the developed procedure for secondary gluteal implant replacement, from intramuscular to submuscular (Figure 1), performed in four private clinical practices between January 2013 and November 2022.

This study included consecutive patients with intramuscular gluteal implants who presented with issues such as implant palpability, capsular contracture, implant hernia, displacement, asymmetry, rippling, implant herniation, or rupture, necessitating secondary surgery. Patients with concomitant gluteal skin ptosis due to weight loss requiring posterior lifting were also included. Patients with a history of sciatic nerve pain or spinal disc herniation, regardless of symptoms, were deemed unsuitable candidates and thus were not eligible for surgery. The study adhered to the ethical principles outlined in the 1975 Declaration of Helsinki, and all patients provided informed consent before undergoing surgery, for their inclusion in the study, and for the use of their images.

### 2.2. Technical Description of the Procedure: The Safe Hybrid Bridge Technique

A supplementary video illustrating the complete technique described in this manuscript has been included to provide a detailed visual representation and enhance procedural understanding (Video S1).

**Figure 2:**
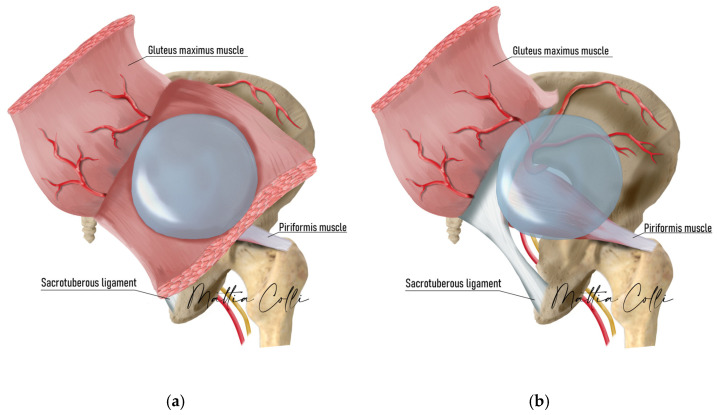
Difference in muscle thickness and implant placement depth between intramuscular (**a**) and submuscular (**b**) pockets.

Implant selection was made by carefully palpating the pre-surgery intramuscular pocket, skin thickness, implant volume, tension, and animation of the gluteus maximus muscle during contraction to understand to which extent the gluteal maximus muscle bridge thickness was intact, which depth it had, and if the muscle would be able to cover the new submuscular implant (Figure 2).

For individuals with a thin intergluteal bridge muscle, opting for an implant size between 225 cc and 340 cc is advisable. Alternatively, a larger implant size may be preferable if the intergluteal bridge gluteal muscle has an average to large thickness. The intramuscular bridge of the gluteal maximus muscle refers to the gluteus maximus muscle fibers displaying robust development, delineating the muscle from the implant pocket to the submuscular space (Figure 1). The more the intramuscular implant pocket was superficially localized, the more likely the intramuscular bridge would be trophic, allowing a more voluminous implant replacement in the new submuscular pocket.

With the patient standing in front of us, pushing the intramuscular implant upwards and at the lateral gluteal area near the posterior margin of the iliac crest, where the muscle becomes thinner, the surgeon looks for a possible drift shift of the intramuscular pocket, rendering the implant more superficial and palpable. The implant edge is drawn on the skin as a reference point with an arc line of 4–5 cm, where the superficial area is more extensive, which should be near the superior iliac crest.

**Figure 3:**
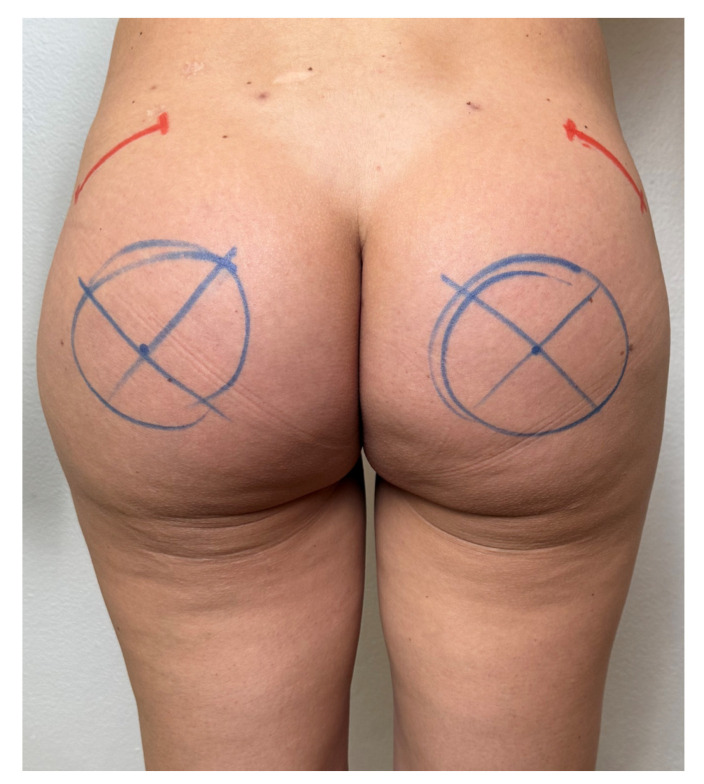
Surgical drawing of planned incisions for submuscular implant placement (blue lines). The red lines show the planned incisions for removing the old implant from the intramuscular pocket.

Prophylactic antibiotic treatment with a third-generation cephalosporin, cefazoline 2 g intravenously, was administered 20 min before skin incision. Markings were made with the patient standing upright, outlining the implant location around the optimal point of maximal buttock projection, considering the position and volume of the intramuscular implant. Given the round base of the prosthesis, the marking resembled a circle around this point (Figure 3). Incisions were carefully made to preserve the intergluteal fold and underlying ligament, according to Gonzales’ intramuscular technique [14]. Robles advised against excessive padding beneath the patient, particularly around the pelvic region, to prevent undue stretching of the gluteal muscles [30,31,32].

New and safe para-sacral scars, measuring 3–4 cm in length, were selected for creating the ideal deep tunnel. For right-handed surgeons, the left incision was made slightly higher (2 cm) to facilitate better blood supply from the contralateral dermal area. Surgery was performed either under general anesthesia, deep sedation, or epidural anesthesia, with the patient lying prone on the operating table in a fully flat position, following the established protocol for primary submuscular augmentation gluteoplasty [30].

Diathermic cutting instruments or bipolar forceps were avoided. Drainage tubes were not used for the submuscular pocket but only for the explantation of the old intramuscular implant.

**Figure 4:**
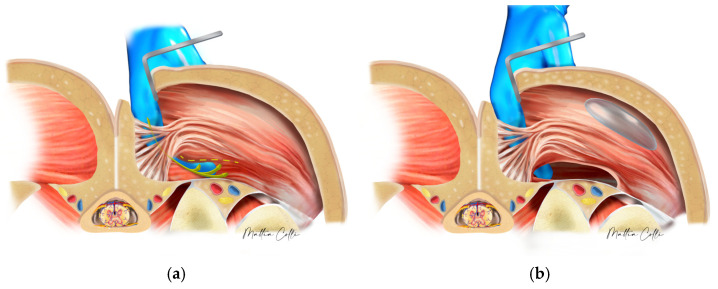
The surgeon must follow the path of least resistance and maintain the depth of the underlying plane, which is performed bluntly using the fingers with an upward sweeping motion (green arrows): (**a**) building the tunnel for the submuscular space; (**b**) keeping all the gluteal maximus muscle fibers in perfect integrity, especially those that will form the protective muscle bridge between the intramuscular and submuscular pocket and the new implant coverage.

The surgeon continued by bluntly opening the subcutaneous tissue from the para-sacral scars with scissors to reach the lateral border of the sacrum, where the fascia of the gluteus maximus muscle is inserted [21,27,33,34,35,36,37,38,39,40,41,42,43,44,45,46]. A small cavity was created by manipulating the muscle tissue without directly encountering the implant or capsule (Figure 4). No other muscle undergoes any surgical digital modification.

**Figure 5:**
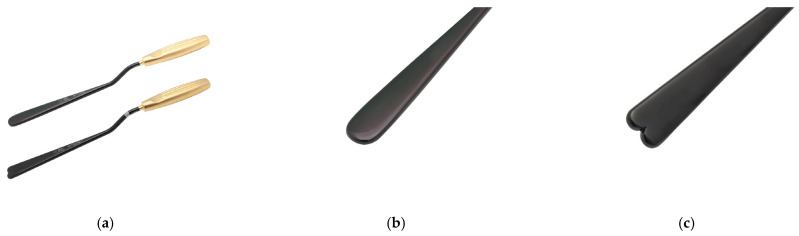
Long spacers are used to enlarge the surgical pocket (**a**). Spacers with a round end are meant to check for the roundness of the surgical pocket (**b**). Spacers with a heart-shaped end are intended to free gluteal muscle adherences (**c**).

Smooth gluteal spacers (Figure 5) were inserted to enlarge the pocket and ensure the muscular bridge between the intramuscular and submuscular pockets remained intact and thick.

The implant was placed in a deep pocket to minimize the risk of extrusion, as muscle contraction would help secure its position. The expansion of tissues was achieved progressively without cutting the fascia or muscle. A dry laparotomic gauze was placed in the submuscular pocket to keep it open while working on creating the exit tunnel from the iliac crest skin [30]. Depending on the volume of the intramuscular implant, a skin incision of 4–4.5 cm for smaller implants (around 200 cc–300 cc) up to 5–6 cm for larger ones was made near the superior iliac crest. In patients requiring a posterior and gluteal lift due to gluteal ptosis from weight loss, direct access could be achieved from the subcutaneous layer.

**Figure 6:**
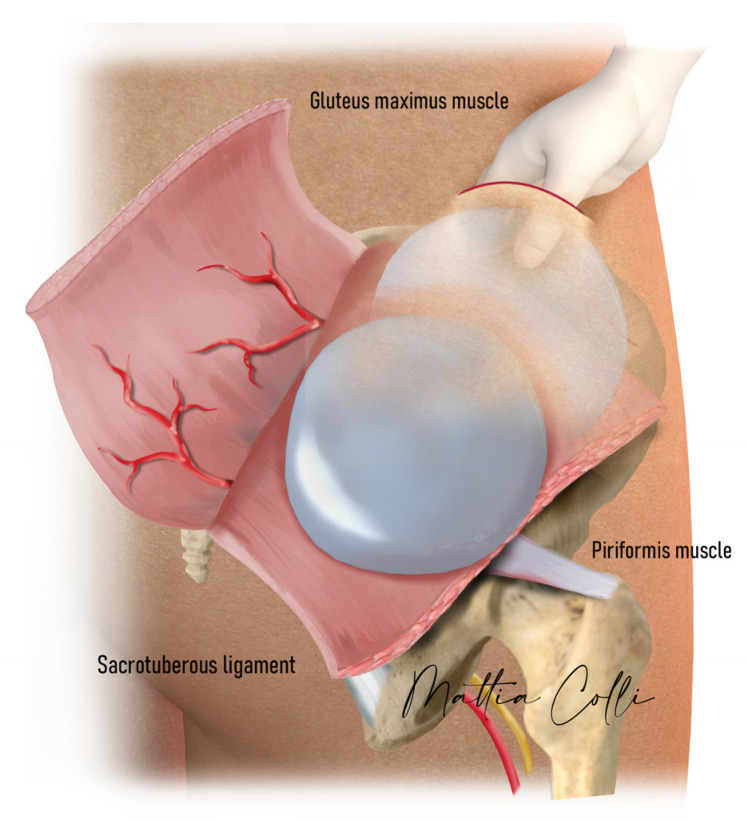
The picture shows the surgeon’s index finger and thumb accessing the intramuscular pocket during implant removal.

**Figure 7:**
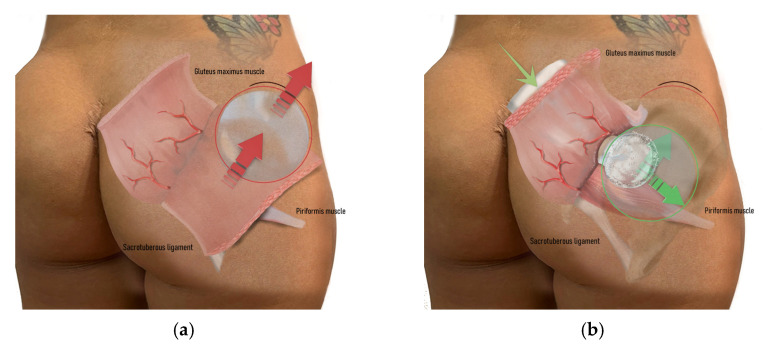
Secondary submuscular gluteal implant replacement. (**a**) Schematic of old implant removal, where the red arrow and circle represent the old, more superficial intramuscular implant being removed. A skin incision at the superior-lateral mark near the superior iliac crest allows the surgeon’s index finger and thumb to access the intramuscular pocket to remove the implant, leaving the muscular bridge between the intramuscular and submuscular spaces intact. (**b**) Schematic of the new implant being inserted with a funnel into the deeper submuscular pocket.

**Figure 8:**
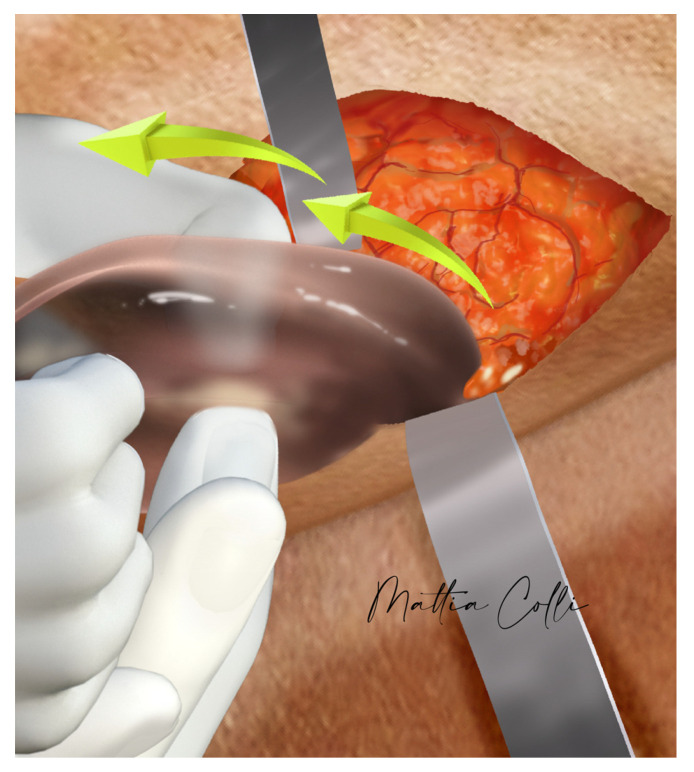
Computer rendering of the surgeon’s fingers accessing the intramuscular pocket and gently removing the implant.

Blunt dissection is continued through the subcutaneous tissue using scissors until reaching the superior anterior fascia of the gluteus maximus muscle. The physician assistant delicately applies upward pressure on the intramuscular implant towards the incision at the skin iliac crest. At this juncture, the capsule surrounding the implant is incised with scissors, enabling the surgeon’s index finger and thumb to enter the intramuscular pocket and gently remove the implant (Figure 6, Figure 7 and Figure 8).

**Figure 9:**
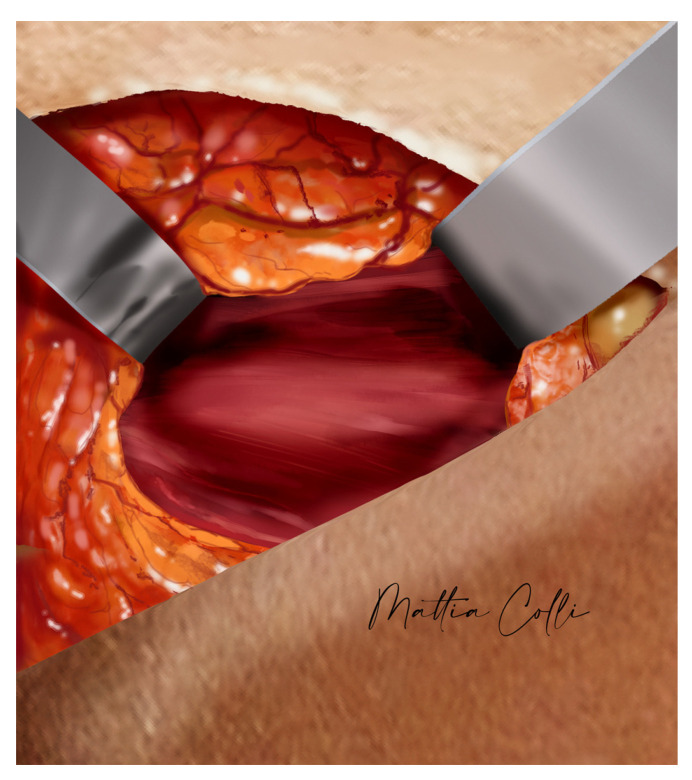
Computer rendering of the intramuscular white capsule integrity that covers the gluteal maximus muscle bridge fibers separating the submuscular pocket.

Using a light retractor or a deep focal light on the head, it is possible to see the intramuscular capsule integrity (Figure 9) that covers the gluteal maximus muscle bridge fibers that separate the submuscular pocket; the thicker the muscle bridge is, the greater the coverage and assurance of esthetic outcomes we achieve during post-surgery recovery. If the gauze is visible from the iliac crest skin access, the muscle bridge has been disrupted, an improper submuscular pocket has been created, or the muscle bridge was too thin to maintain its structural integrity.

Once the intramuscular implant has been removed, serous liquid can be produced in the old pocket until the two capsule walls adhere. Therefore, it is recommended to place a small drain for 2–3 days in the old intramuscular pocket and close the subcutaneous layer with Vicryl 3–0 and intradermic skin suture with Nyon 4–0 to be removed 12 days after surgery. The capsule is not removed; being very thin, it is not noticeable and does not influence the esthetic result. At this point, the surgeon can work on the other side with dry laparotomic gauze placed into the first submuscular pocket. Once the laparotomy gauze is removed, the surgeon checks its cleanliness and the submuscular pocket: the cavity can be perfected by introducing a finger; in particular, in this phase, it is recommended to use the medium finger, which is the longest, and smooth gluteal spacers.

The authors prefer implants featuring a round base, smooth surface, and cohesive silicone gel filling (Polytech^®^ Round POLYsmooth^®^ implants (POLYTECH^®^ Health & Aesthetics, Dieburg, Germany) were utilized in this series). A funnel proves highly beneficial to facilitate implant insertion (we employed Keller-Funnel 2^®^ (Allergan Aesthetics, Irvine, CA, USA) or EzFan^®^ (Kims Med Co., Ltd., Gwangju, Republic of Korea)), enabling implant placement through a small skin incision using a no-touch technique (Figure 7b). In the para-sacral area, the small fascia opening remains untouched, and skin closure is conducted in two layers: the deep subcutaneous layer is brought together with a long, 1⁄2 circle needle using a 0 braided absorbable suture; the dermal plane is closed with a continuous intradermal suture, proceeding from bottom to top, using a few 3–0 braided absorbable monofilaments.

Postoperatively, the patient should be supine for one hour in a semi-sitting position with a slightly elevated and bent backrest, supported by one or two pillows at the level of the popliteal fossa. Subsequently, the patient is allowed to stand, walk, and sit cautiously, avoiding leaning forward or bending excessively at the waist; bodyweight squat movements are prohibited for at least three months. Patients are typically discharged a few hours after surgery (usually 3–5 h) and provided with tight compression garments such as Tensoplast^®^ to maintain compression from the top of the buttocks downward.

Upon discharge, patients receive oral prescriptions for pain control medications (e.g., paracetamol 500 mg/codeine 30 mg twice daily and ketorolac oral drops as needed) and a muscle relaxant like diazepam. Steroids may be administered later if sciatic nerve irritation occurs.

## 3. Results

We performed the described secondary gluteal implant replacement surgery on 108 patients, including 96 females (88.9%) and 12 males (11.1%). The mean age of the patients was 40.0 ± 10.5 years, ranging from 20 to 69 years. Surgery duration ranged from 60 to 90 min, with a median of 75 ± 15 min. The mean preoperative body mass index (BMI) was 21.3 ± 2.1 kg/m^2^ (range 15–31 kg/m^2^). The mean surgical time was 58.7 ± 21.8 min (range 40–180 min). A concomitant liposuction in the buttock area was performed in 47 cases (43.5%) with a mean fat removal of 411.9 ± 168.6 cc (range 200–960 cc). The average size of used implants was 277.3 ± 70.1 cc (range 225–560 cc).

Complications were observed in 17 out of 108 patients (15.7%) (Table 1). Some patients experienced more than one complication, resulting in overlapping events. The most prevalent was sciatic pain lasting over 10 days: this was experienced by eight patients (7.4%) but resolved spontaneously during follow-up without the need for intervention. Hematoma occurred in six cases (5.6%), and delayed wound healing in two cases (1.9%). Incision dehiscence resolved spontaneously and did not require surgical repair, and no implant exposures were detected in this series. There were no cases of complete sciatic nerve damage (palsy). Temporary and partial alterations in sensitivity, tingling, and difficulty in hip movement and leg raising occurred in four patients (3.7%) and resolved spontaneously within 3 to 5 days.

Asymmetry occurred in four patients (3.7%), with one implant slightly higher than the contralateral. Implant flipping (rotation from front to back) was observed in seven patients (6.5%), unilaterally in all cases.

We performed an exploratory multivariable analysis to investigate potential associations between complications and selected risk factors. Specifically, we assessed the impact of BMI (OR (odds ratio), 0.87; 95% CI (confidence interval), 0.64 to 1.20; *p* = 0.369), cigarette smoking (OR, 0.55; 95% CI, 0.18 to 1.68; *p* = 0.291), age (OR, 1.03; 95% CI, 0.98 to 1.10; *p* = 0.222), and implant volume (OR, 1.00; 95% CI, 0.99 to 1.01; *p* = 0.882). No statistically significant associations were found, although the analysis is underpowered and should be interpreted with caution due to the small number of events.

**Figure 10:**
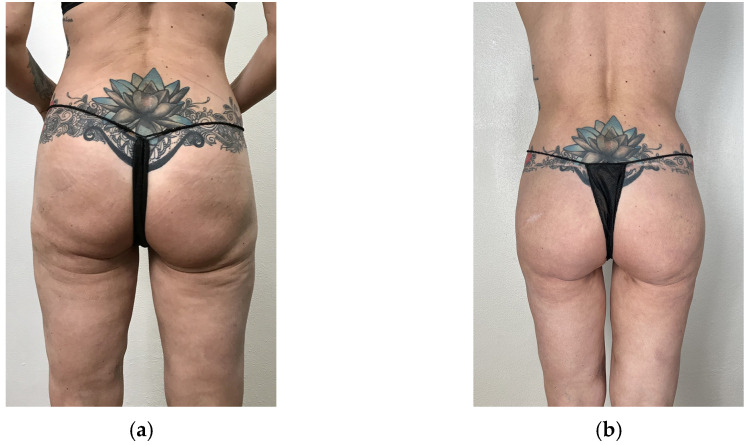

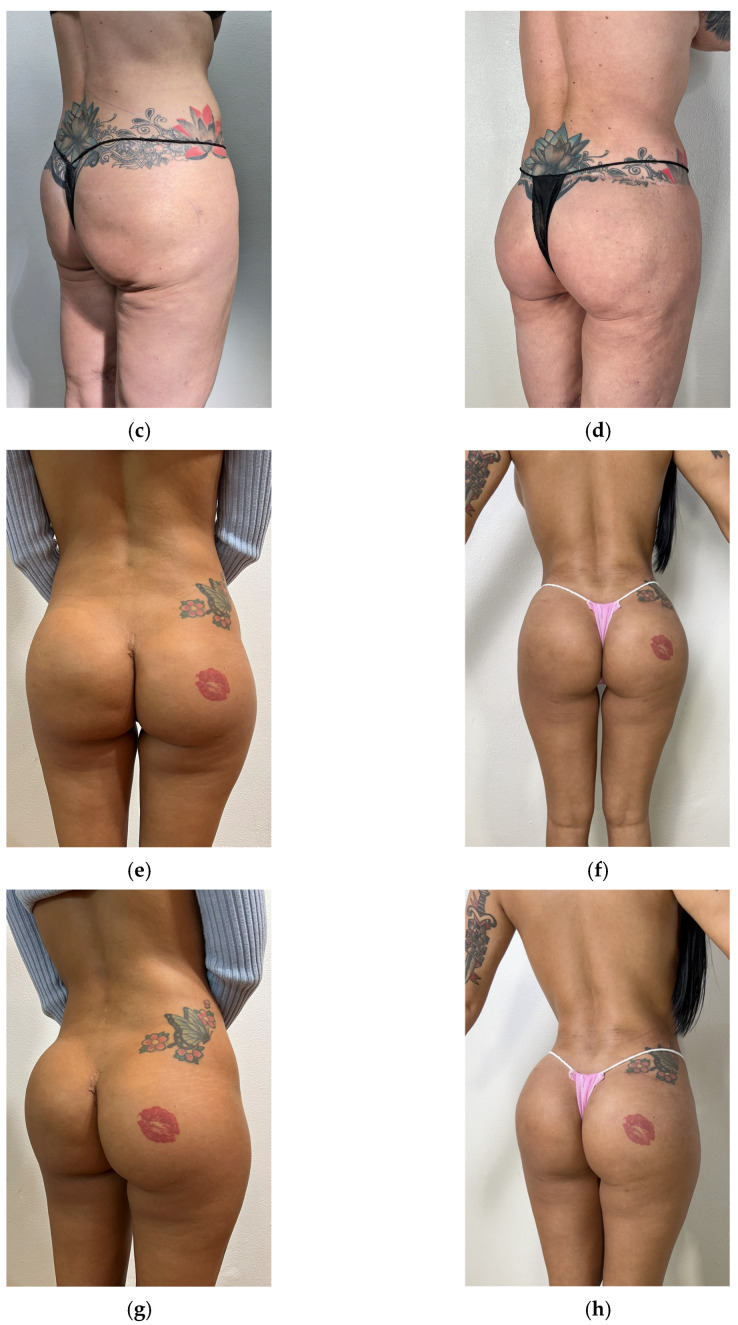
Results in two female patients who replaced the old intramuscular implant with a new implant in the submuscular pocket. Patient 1 (**a**–**d**) also performed a posterior and gluteal lift and a medial thigh lift. Patient 2 (**e**–**h**) only performed secondary submuscular gluteal implant replacement. The first column (**a**,**c**,**e**,**g**) shows the patients before surgery, and the second column (**b**–**h**) shows the patients after surgery.

Figure 10 shows pre- and postoperative pictures of two representative women who underwent our secondary implant replacement surgery.

Patients undergoing secondary gluteal replacement express a high level of satisfaction; thanks to the deep positioning of the implants, they remain invisible and almost imperceptible in all positions of the buttock area, offering no subtle indication that surgery has been performed. Despite initial postoperative discomfort, which includes difficulty sitting for extended periods during the early recovery phase and requiring greater attention compared to primary procedures, particularly in the following months, no patient in this series expressed a desire for implant revision or replacement during the postoperative extended follow-up period of a minimum of 6 months.

## 4. Discussion

The intramuscular pocket, with its varying muscle thickness separating the implant from subfascial and submuscular planes, offers better implant concealment than suprafascial placement [21,43,47]. However, it poses challenges such as moderate dislocation, muscle atrophy, and implant migration due to muscle contraction, potentially leading to poor implant coverage in some clinical cases, particularly in the presence of subcutaneous fat lamination, weight loss, and skin laxity.

The dual-plane pocket, combining a submuscular cranial half and an intramuscular caudal half, presents challenges in achieving symmetrical dissection of the inferior intramuscular plane within anatomical gluteus maximus muscles [48]. Submuscular undermining should be meticulously performed in a deep plane between the medius and maximus gluteus muscles.

The posterior iliac approach allows for precise and atraumatic removal of the old implant through a small tunnel where the muscle is thinner, making it easier to palpate and locate the implant without disrupting the central muscle structure. In addition, this approach avoids further widening of the intergluteal scar or altering the natural intergluteal fold, reduces tension on the scar, and remains well hidden laterally under clothing.

Complications of gluteal implant surgery, such as seroma, wound dehiscence, implant extrusion, and visibility or palpability of the implant, can be significantly reduced or prevented by placing the implant submuscularly [49,50]. However, this approach has been less favored by some surgeons due to concerns about damaging deep structures like the sciatic nerve or gluteal vessels [44] and the difficulty in identifying the submuscular plane, which is thought to be smaller than the intramuscular plane and in direct continuity with the sciatic nerve. Indeed, a comprehensive understanding of the sciatic nerve’s anatomy is crucial when intervening in the gluteal region, even during gluteal intramuscular injections, which can lead to sciatic neuropathy [35,36]. In our innovative surgical technique, the submuscular space presents intact tissues during the dissection. The goal of delivering the intramuscular implant through an alternative surgical access is precisely to preserve the tissue bridge (or flap) as intact as possible, thereby reducing the risk of seromas and implant dislocation to rates comparable to those seen in primary submuscular gluteoplasty [30]. Moreover, despite the deeper implant positioning and absence of drainage in the submuscular pocket, there were no cases of complete sciatic nerve damage (palsy), a concern that has been raised but is not supported in the relevant literature [14]. Submuscular implant placement is a secure method for buttock augmentation. Regardless of the implant size, this approach ensures optimal coverage, protection, and concealment of the implant, making it nearly undetectable visually and by touch [30]. In our clinical experience, we did not observe increased implant palpability or visibility in the lateral gluteal region, even in patients with lower BMI. Careful expansion of the submuscular pocket using long, smooth spacers and blunt digital dissection helped ensure symmetrical lateral extension of the pocket. Additionally, intraoperative implant selection was tailored to each patient’s muscle bridge thickness, muscle volume, tissue coverage, and subcutaneous tissue thickness, further reducing the risk of lateral visibility or contour irregularity. In patients with low BMI, smaller-volume implants were selected compared to those with higher BMI.

The authors opted against using implants with anatomical shapes due to concerns about proper orientation, potential twists, and tissue coverage thickness [32].

In this series, the most common early complication following secondary surgery was wound dehiscence and delayed healing, typically resolving within 28 days. The incidence of wound dehiscence is consistent with rates observed in other studies employing similar techniques [14,30,45,46]. In general, the complication rate was highly consistent with the latest published report from our team and literature reviews [14,30,35,51], confirming that the deep submuscular surgical gluteal technique offers a better esthetic result but does not eliminate potential complications.

Our exploratory multivariable analysis did not reveal any significant association between risk factors and complications. This could be explained by the multifactorial nature of postoperative outcomes. However, given the limited number of events, this analysis lacks statistical power and should be interpreted cautiously. Future studies with larger cohorts are needed to better evaluate potential risk factors.

There were also a few cases of implant flipping. This flipping may be induced by the presence of periprosthetic fluid, which facilitates implant rotation; even in small pocket-size cases, the forces exerted during sitting and movement could potentially enlarge the pocket and lead to flipping.

Although our study does not include a comparative group treated via the conventional intergluteal approach, the observed complication rates are in line with or lower than those reported in the literature for secondary gluteal augmentation procedures using standard access routes [27]. The posterior iliac access, by minimizing trauma to the central gluteal structure and preserving the muscle bridge, may contribute to enhanced implant stability. We acknowledge the absence of a direct comparison as a limitation of our current study. Future prospective studies including control groups would be valuable to confirm whether the posterior approach offers a statistically significant advantage in reducing complications like displacement and flipping.

Muscle fiber dissection was not performed, resulting in limited fluid collection; thus, drainage is not required. Only in two clinical cases did the muscular bridge between the intramuscular and the submuscular pockets break by joining the two anatomic pockets, with no esthetic result for the patient. This can occur because either the bridge is too thin, the submuscular pocket is not adequately perfected, or the volume chosen for the new implant is too big for the covering bridge’s muscular tissue.

Potential sciatic nerve irritation was addressed with oral steroids, with a regimen of 60 mg of prednisone daily for 2 days, gradually tapered down by 10 mg per day thereafter. This irritation could be attributed to increased pressure in the initial postoperative period and the proximity of the submuscular implant to the nerve.

A limitation of this study is inherent in its study design. This is a real-life, retrospective observational study that collects the experiences of the private practices of the four authors over an extended period. As such, it carries potential biases since it relies on existing records and has limited generalizability. However, data collection in our practices is very thorough; therefore, it is unlikely that it was incomplete. Additionally, the study design inherently lacks randomization and controlled conditions. However, we did not aim to claim non-inferiority, but our scope was to share our safe technique. Furthermore, while we collected BMI and intraoperative assessments of soft tissue thickness, we did not stratify complication rates (e.g., implant palpability, contour deformities) according to patient body habitus. This is an important consideration, particularly in low-BMI patients, and should be addressed in future studies to better predict esthetic outcomes and refine patient selection criteria.

Also, we did not stratify or correlate preoperative complaints with individual postoperative outcomes in a structured manner. This level of detail was beyond the scope of our retrospective study design. We acknowledge that a prospective analysis with predefined outcome measures and esthetic scoring would be valuable for assessing how well each specific concern is addressed by the Safe Hybrid Bridge Technique. We encourage future studies to explore this approach.

Our follow-up times were at least 6 months for all our patients, which aligns with recent literature on similar procedures [49]. Future studies are required to assess if our technique is the best option for gluteal augmentation, including more objective satisfaction outcome measures, and to follow up with patients for longer (>12 months) to ascertain that the procedure is safe over extended periods, and that esthetic results and patient satisfaction last. However, in the long period when the technique has been used in our practices, no patient has returned with complaints about the replaced implant.

## 5. Conclusions

To improve esthetic gluteal augmentation coverage, we have considered the surgical techniques presented by Gonzalez [14] and Mendieta [46] and the new submuscular technique by Colli and Petit [30]. In this work, the submuscular gluteal replacement and augmentation with the Safe Hybrid Bridge Technique give patients a second chance for improved buttock esthetic results. The procedure fulfills the aspirations of patients desiring a more natural appearance and a discreetly planned augmentation. The submuscular technique can often be a second esthetic change for those unsatisfied with their intramuscular implant.

## Figures and Tables

**Table 1:** Complications observed in treated patients.

Complication	Number of Patients	Percentage of Total (N = 108)
All *	17	15.7%
Sciatic pain lasting >10 days	8	7.4%
Implant flipping	7	6.5%
Hematoma	6	5.6%
Temporary alteration in sensitivity	4	3.7%
Asymmetry	4	3.7%
Delayed wound healing	2	1.9%

* Each patient could have more than one complication.

## Data Availability

The original contributions presented in this study are included in the article/Supplementary Material. Further inquiries can be directed to the corresponding author.

## Supplementary Materials

The following supporting information can be found at: https://doi.org/10.5281/zenodo.14886481 (accessed on 18 February 2025), Video S1: The Safe Hybrid Bridge Technique.
